# Human Microglia Extensively Reconstitute in Humanized-BLT Mice With Human Interleukin-34 Transgene and Support HIV-1 Brain Infection

**DOI:** 10.3389/fimmu.2021.672415

**Published:** 2021-05-21

**Authors:** Jianshui Zhang, Saroj Chandra Lohani, Yilun Cheng, Tao Wang, Lili Guo, Woong-Ki Kim, Santhi Gorantla, Qingsheng Li

**Affiliations:** ^1^ School of Biological Sciences, University of Nebraska-Lincoln, NE, United States; ^2^ Nebraska Center for Virology, University of Nebraska-Lincoln, NE, United States; ^3^ Department of Pharmacology and Experimental Neuroscience, University of Nebraska Medical Center, Omaha, NE, United States; ^4^ Department of Microbiology and Molecular Cell Biology, Eastern Virginia Medical School, Norfolk, VA, United States

**Keywords:** microglia, interleukin-34, NOG mice, Hu-BLT mice, HIV-1

## Abstract

Humanized bone marrow-liver-thymic (hu-BLT) mice develop a functional immune system in periphery, nevertheless, have a limited reconstitution of human myeloid cells, especially microglia, in CNS. Further, whether bone marrow derived hematopoietic stem and progenitor cells (HSPCs) can enter the brain and differentiate into microglia in adults remains controversial. To close these gaps, in this study we unambiguously demonstrated that human microglia in CNS were extensively reconstituted in adult NOG mice with human interleukin-34 transgene (hIL34 Tg) from circulating CD34+ HSPCs, nonetheless not in hu-BLT NOG mice, providing strong evidence that human CD34+ HSPCs can enter adult brain and differentiate into microglia in CNS in the presence of hIL34. Further, the human microglia in the CNS of hu-BLT-hIL34 NOG mice robustly supported HIV-1 infection reenforcing the notion that microglia are the most important target cells of HIV-1 in CNS and demonstrating its great potential as an *in vivo* model for studying HIV-1 pathogenesis and evaluating curative therapeutics in both periphery and CNS compartments.

## Introduction

Microglia, the resident macrophages in the central nervous system (CNS), are the key resident immune cells to maintain neuronal homeostasis, defend against infections, and are associated with the pathogenesis of many neurodegenerative diseases (1–3). The ontology of adult brain microglia has been debated for a long time. The consensual view to date is that microglia in CNS is the seeding results of primitive hematomyeloid precursor cells from yolk sac and aorta-gonad-mesonephros region in early embryo life and proliferation *in situ* thereafter (4–8). However, multiple studies also showed that bone marrow derived cells can enter the brain and differentiate into microglia in adults (9–11).

Humanized mice (hu-mice) with a human immune system have been extensively used in investigating the ontology of immune cells, immunopathogenesis of human specific pathogens, and evaluating therapeutics as preclinical small animal models (12–15). Hu-mice generated by engrafting human CD34+ hematopoietic stem and progenitor cells (HSPCs) in neonatal life can reconstitute macrophages in the meninges and perivascular spaces, but rarely in the parenchyma of brain (16, 17). Similarly, humanized bone marrow-liver-thymic (hu-BLT) mice engrafted with human fetal liver and thymic tissues and HSPCs at adults develop a functional immune system in periphery but have a limited reconstitution of human myeloid cells, especially microglia, in CNS (18). We previously demonstrated that human interleukin-34 transgenic (hIL34-Tg) NOG mice engrafted intrahepatically with CD34+ HSPCs at birth significantly reconstituted microglial-like cells in the CNS (19). However, it remained unknown whether adult hIL34-Tg NOG mice could also reconstitute human microglia in CNS until this study. The hu-BLT mice are the best hu-mice in terms of human immune reconstitution, as they are engrafted with human fetal thymic tissues in addition to human liver tissues and liver derived CD34+ HSPCs where human T cells can receive differentiation and selection education in human thymic tissues (20, 21). This study has been poised to address two questions using the hu-BLT hIL34-Tg NOG (hu-BLT-hIL34) mouse model. First, we wanted to investigate whether human parenchymal microglia in the CNS could be reconstituted in adult hIL34 Tg mice from circulating myeloid precursor cells derived from CD34+ HSPCs. This is a fundamental question regarding the origin of human microglia in CNS at adults. The second question was to test the susceptibility of the reconstituted human microglia to HIV-1 infection.

Using this unique system and by comparing two types of hu-BLT mice with and without hIL34 Tg received the same human donor tissues, we unambiguously demonstrated that human microglia in CNS can be extensively reconstituted in adult hIL34 Tg NOG mice but no in NOG mice, which provides strong evidence that human CD34+ HSPCs can enter adult brain and differentiate into microglia in the CNS in the presence of hIL34. Further, the human microglia in the CNS of hu-BLT-hIL34 mice are susceptible to HIV-1 infection, which reenforced the notion that microglia are the most important target cells of HIV-1 in CNS and demonstrated its great potential as an *in vivo* model for studying HIV-1 pathogenesis and evaluating curative therapeutics in both periphery and CNS compartments.

## Method

### Ethics

All methods associated with animals described in this study were conducted in accordance with the Institutional Animal Care and Research Committee (IACUC) approved protocols at the University of Nebraska-Lincoln (UNL) and University of Nebraska Medical Center (UNMC).

### hIL34-Tg NOG and NOG Mice

NOG (NOD.Cg-*Prkdc^scid^ Il2rg^tm1Sug^*/JicTac) mice of 6-8-week-old were purchased from Taconic Biosciences (Rensselaer, NY 12144, United States) and housed at the UNL Life Sciences Annex under specific-pathogen-free conditions. The hIL34-Tg NOG mice were bred at UNMC by pairing hIL34-Tg mouse with NOG mouse of opposite gender. The offspring were genotyped after three weeks of age by obtaining DNA from the tail snipping. Genotyping was performed for hIL34 as described previously using real-time polymerase chain reaction (19). Mice positive for hIL34 were transferred to UNL to generate hu-BLT mice.

### Generation of hu-BLT-hIL34 and hu-BLT Mice

**Figure 1:**
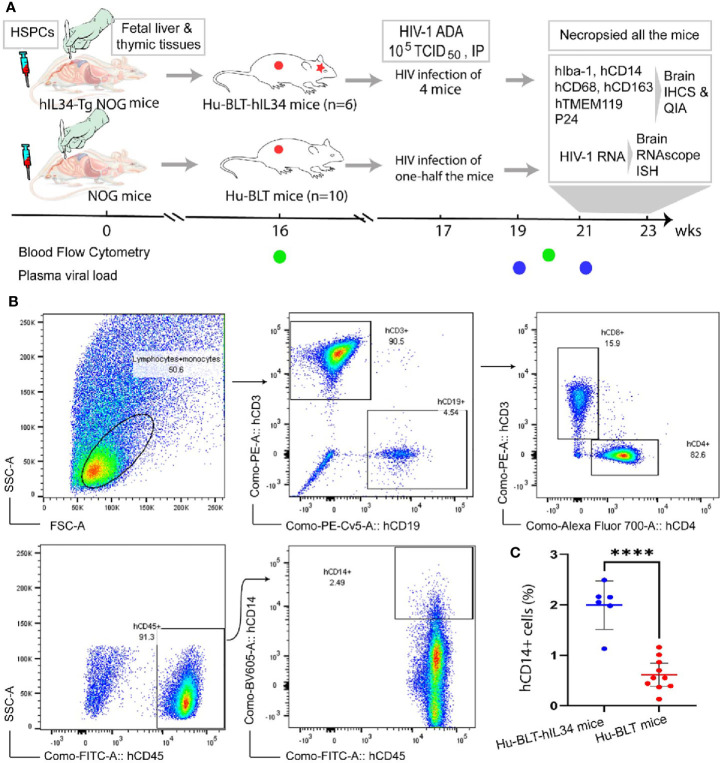
Experimental timeline and Flow Cytometric evaluation of human immune cells reconstitutions in the peripheral blood of hu-BLT-hIL34 and hu-BLT mice. **(A)** Experimental design and timeline for the humanization of hIL34 transgenic mice and NOG mice by engrafting with human CD34+ hematopoietic stem and progenitor cells (HCSPs) and human fetal liver and thymic tissues, HIV-1 Ada infection of some mice in each group, and the evaluation of human myeloid and microglial cell reconstitution and HIV-1 infection in the CNS of euthanized mice using immunohistochemical staining (IHCS) and RNAscope *in situ* hybridization (ISH). **(B)** The representative flow cytometric dot plots of the peripheral blood mononuclear cells of a hu-BLT-hIL34 mouse (#1703) at the 4 months after transplantation, which were gated with hCD45+, hCD3+, hCD9+, and hCD14+ cells. **(C)** The percentage of hCD14+ myeloid cells in the peripheral blood of hu-BLT-hIL34 mice is significantly higher than that hu-BLT mice at the 4 months post transplantation. Each symbol represents an individual mouse, hu-BLT-hIL34 mice n=6, hu-BLT mice n=10; ****p < 0.0001.

To investigate human microglia reconstitution in the CNS of hu-BLT-hIL34 mice, both hu-BLT-hIL34 and hu-BLT mice were generated from adult hIL34-Tg NOG and NOG mice as we previously reported (22, 23) (**Figure 1A**). Briefly, 6-to-8-week-old adult hIL34-Tg NOG mice and NOG mice received sublethal irradiation at the dose of 12 cGy/gram of body weight with an RS2000 X-ray irradiator (Rad Source Technologies). Mice were surgically engrafted with a sandwich of two pieces of human fetal liver and one piece of thymic tissue fragments under the murine left renal capsules, of which human fetal livers and thymus tissues were procured from the Advanced Bioscience Resources. Within 6 hours after surgery, a total of 2.3 × 10^5^ fetal liver derived CD34^+^ HSPCs in 200 ul volume was injected through tail vein. At 16 weeks post engraftment, the immune reconstitution in the peripheral blood was assessed using flow cytometry as described below.

### Human Brain Tissues

To compare the human microglia from hu-BLT-hIL34 mice with humans, ethically sourced human autopsy cerebral cortex tissues from a deidentified individual of HIV-1 negative with no registered medical complications were obtained from the NIH Neuro BioBank (https://neurobiobank.nih.gov/).

### Multicolor Flow Cytometry

Multicolor flow cytometry was conducted as previously reported (22, 23) for assessing the human immune reconstitution of peripheral blood mononuclear cells (PBMCs) at 16- and 20-weeks post transplantation (**Figure 1B**). Briefly, peripheral blood was collected from a great saphenous vein into the ethylenediaminetetraacetic acid (EDTA)-containing vial (BD Microtainer, Franklin Lakes, NJ, USA). Red blood cells were lysed using fluorescence activated cell sorting (FACS) lysing solution (BD Biosciences, USA). The PBMCs were resuspended in a FACS buffer (2% FBS in phosphate-buffered saline) and incubated with a cocktail of following monoclonal antibodies against human immune cell markers at 4°C for 30 min: anti-hCD45-fluorescein isothiocyanate (FITC), anti-hCD3-Phycoerythrin (PE), anti-hCD19-Phycoerythrin-Cyanin 5 (PE-Cy5), anti-hCD4-Alexa Fluor 700 (AF700), anti-hCD8-allophycocyanin-Cyanin 7(APC-Cy7) and anti-hCD14-Brilliant Violet 605 (BV605). All above antibodies and isotype control antibodies were obtained from BD Biosciences, USA. The stained cells were washed with FACS buffer and fixed with 4% paraformaldehyde. Raw data were acquired by FACSAria III (BD Biosciences) and analyzed with FlowJo version 7.6.4 (TreeStar). Statistical analysis was performed using GraphPad Prism 7 (GraphPad Software). Two groups of female hu-BLT-hIL34 (n=6) and hu-BLT mice (n=10) with good human immune cell reconstitutions in the peripheral blood (**Table 1**) were selected for this study.

**Table 1:** The blood human immune cells reconstitutions of hu-BLT-hIL34 and hu-BLT mice.

Group	Mouse ID	hCD45 (%)	hCD3+ (%)	hCD8+ (%)	hCD4+ (%)	hCD19+ (%)	hCD14+ (%)
Hu-BLT-hIL34	1699	92.10	88.90	14.70	83.80	7.76	1.98
	1703	91.30	90.50	15.90	82.60	4.54	2.49
	1705	16.60	94.60	49.00	49.00	0.88	2.05
	1707	85.80	99.60	10.80	88.40	0.02	1.13
	1708	83.50	91.20	11.80	87.00	4.93	2.16
	1709	7.62	87.80	55.00	41.00	5.79	2.15
Mean		62.82	92.10	26.20	71.97	3.99	1.99
SD		39.52	4.35	20.16	21.14	2.97	0.46
Hu-BLT	1717	83.80	22.40	47.70	49.50	67.80	1.16
	1718	89.50	84.30	19.80	78.50	13.20	0.37
	1720	42.90	94.70	20.80	78.20	3.14	0.44
	1721	78.90	99.50	11.20	87.90	0.02	0.13
	1722	56.30	23.90	46.60	51.40	69.70	0.70
	1723	89.10	64.20	24.30	73.60	32.70	0.55
	1724	70.30	97.60	15.50	83.80	1.24	0.86
	1726	64.70	54.70	72.10	26.50	31.00	1.01
	1728	94.30	81.40	18.20	80.10	15.50	0.39
	1729	93.30	83.60	17.30	80.90	13.10	0.55
Mean		76.31	70.63	29.35	69.04	24.74	0.62
SD		17.32	28.71	19.56	19.81	25.74	0.32

### HIV-1 Infection and Measurement of HIV-1 Plasma Viral Loads

To investigate the infectivity of HIV-1 in parenchymal human microglial cells in the CNS, 4 mice (# 1703, 1705, 1708 and 1709) of the hu-BLT-hIL34 mice group and 5 mice (# 1720, 1723, 1724, 1726, and 1728) in the hu-BLT mice group were randomly selected (**Table 1**) and intraperitoneally inoculated with the 10^5^ tissue culture infectious dose 50 (TCID_50_) of macrophage-tropic HIV-1 ADA in 200μl volume (obtained through the NIH AIDS Reagent Program, Division of AIDS, NIAID, NIH: HIV-1 ADA Virus from Dr. Howard Gendelman). At 2- and 4-weeks post HIV-1 inoculation, HIV-1 plasma viral load (pVL) in copies/ml was determined by real-time RT-PCR using our previously published protocol (24). Briefly, viral RNA was extracted from the plasma using QIAamp ViralRNA minikit (Qiagen) as recommended by the manufacturer and quantified using C1000 ThermalCycler and the CFX96 Real-Time system (Bio-Rad). For 20 µl qRT-PCR, 5 µl of extracted viral RNA, TaqMan Fast Virus 1-Step master mix (Life Technologies) and following primers and probe combination (IDT, USA) were used: Forward Primer, GCCTCAATAAAGCTTGCCTTGA; Reverse Primer, GGGCGCCACTGCTAGAGA; Probe,/56-FAM/CCAGAGTCA/ZEN/CACAACAGACGGGCACA/3IABkFQ/.

### Euthanasia of All the Mice for Evaluating Human Myeloid Cell Reconstitution and HIV-1 Infection in the CNS

After two consecutive positive results of HIV-1 pVL at 2- and 4-weeks post HIV-1 inoculation, which is equivalent to the 21- to 23-weeks post transplantation, all the mice, including HIV-1 infected and non-inoculated subgroups from the hu-BLT-hIL34 and hu-BLT mice groups, were euthanized for analyzing human myeloid cell reconstitutions and HIV-1 infections in the CNS (**Figure 1A**). Whole brain was dissected out during necropsy and sliced coronally into 5 parts at 4 mm interval using a young mouse brain slicer (Cat# BSMYS001-1, Zivic Instruments, Pittsburgh, PA, USA). The brain tissues and other tissues including spinal cord, spleen, lymph node, jejunum and ileum were collected and fixed in SafeFix™ II (Cat# 042600, Fisher Scientific, USA) at room temperature for 6 hours and embedded in paraffin.

### Immunohistochemical Staining (IHCS)

To evaluate the phenotype, morphology and distribution of the reconstituted human myeloid cells in the CNS, IHCS was conducted by following our previously published protocol with slight modifications (25). Briefly, antigen retrieval of 6-μm thick tissue sections were performed in 0.1mM citrate buffer (PH 6.0) by heating at 98°C for 15 mins. The following primary antibodies were used for detecting macrophage/microglial cells: rabbit monoclonal-antibody (mAb) to human ionized calcium-binding adaptor molecule 1 (hIba-1, EPR6136-2 clone, Cat# ab221933, 1:500; Abcam, USA), rabbit polyclonal antibody to human TMEM119-C-terminal (hTMEM119, Cat# ab185333, 1:500; Abcam, USA), rabbit mAb to human CD14 (hCD14, EPR3653 clone, Cat. # 133335, 1:1000; Abcam, USA), mouse mAb to human CD68 (hCD68, PG-M1 clone, Cat# MS-1808-S1, 1:100; Thermoscientific, USA), mouse mAb to human CD163 (hCD163, 10D6 clone, Cat# NCL-L-CD163, 1:100; Leica Biosystems, USA). For HIV-1 detection, mouse mAb to HIV-1 gag p24 (Kal-1 clone, Cat# M0857, 1:10, Dako, USA) was used. Mouse or rabbit IgG isotype control antibodies were used as negative control. Dako EnVision+ system-HRP labelled polymer anti-rabbit kit (Code K4002, Dako, USA) or anti-mouse kit (Code K4000, Dako, USA), and the Betazoid DAB Chromogen Kit (Cat# BDB2004, BioCare Medical, USA) were used for signal detection and visualization. The cell nuclei were counterstained with Mayer’s hematoxylin. The stained tissue sections after completion of IHCS were digitized with Aperio CS2 Scanscope and the quantitative image analysis of positive cells was conducted using a positive pixel count algorithm in Aperio’s Spectrum Plus analysis program (version 9.1; Aperio ePathology Solutions) as previously reported (26).

### HIV-1 Viral RNA Detection Using RNAscope *In Situ* Hybridization (ISH)

HIV-1 viral RNA (vRNA) in the brain tissues were detected using RNAscope ISH according to our previously published protocol (26). Briefly, HIV-1 antisense probes of RNAscope^®^ ISH probe-V-HIV1-clade B (Cat# 416111) and RNAscope^®^ 2.5 HD assay-Red kit were used. The RNAscope^®^ probe-DapB (Cat# 310043) was used as a negative control. All the reagents above were purchased from the Advanced Cell Diagnostics, Inc.

### Combined RNAscope ISH With IHCS

To determine the cell types of HIV-1 vRNA+ cells in the CNS, a combined RNAscope ISH and IHCS method was used as reported (26). Briefly, after the completion of RNAscope ISH for HIV-1 vRNA and digitization of the whole tissue section, the slides were soaked in xylene overnight to remove the coverslip and the tissue section was rehydrated and subjected to IHCS using the rabbit mAb to hIba-1 (clone# EPR6136-2, Cat# ab221933, 1:500; Abcam, USA) as the primary antibody as described in the IHCS section above. Rabbit IgG isotype control antibody was used as negative control.

## Results

### Human Myeloid Cell Reconstitution in the CNS

**Figure 2:**
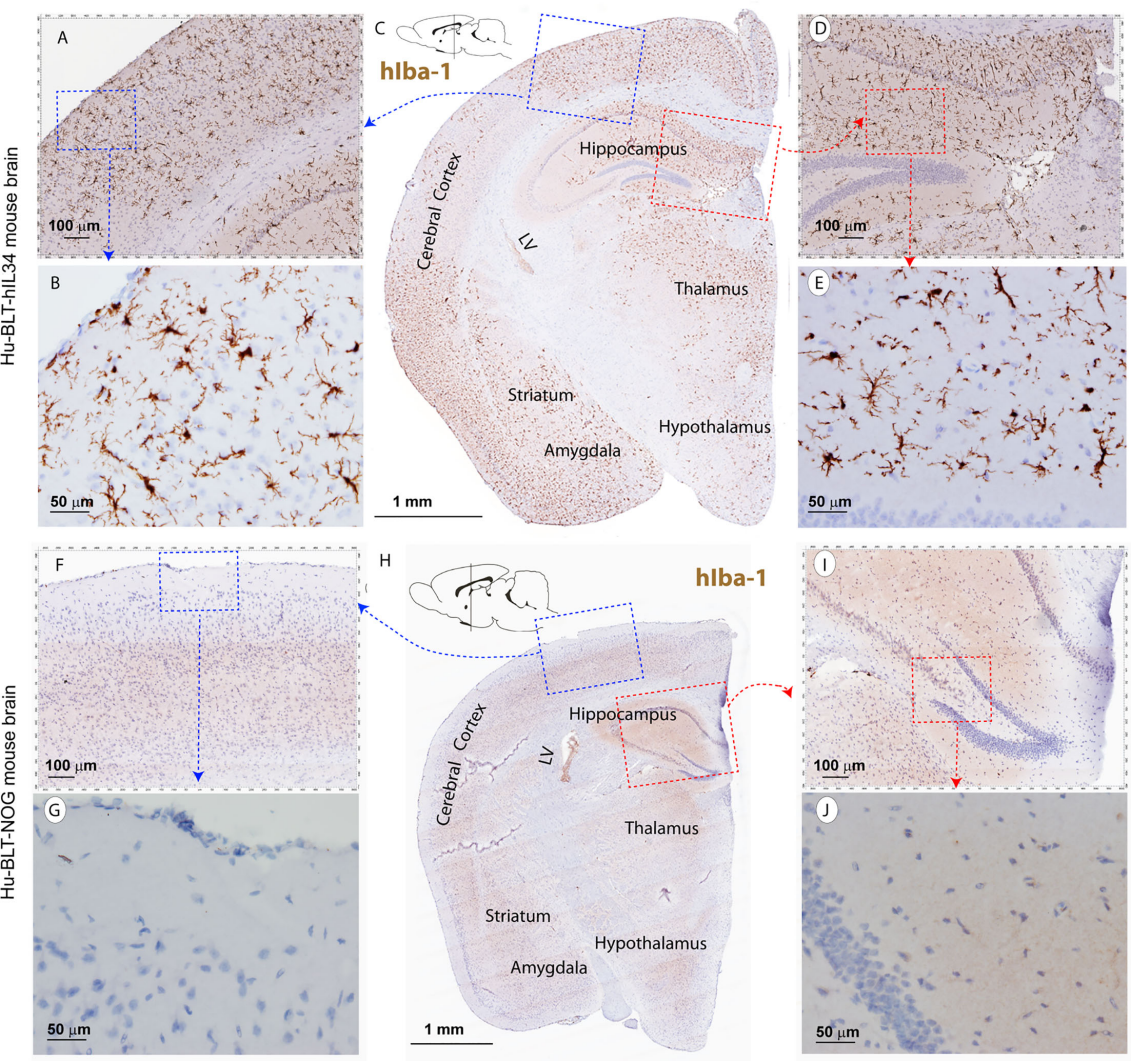
The hIba-1+ myeloid cell reconstitutions in the brain tissues of hu-BLT-hIL-34 and hu-BLT mice. The upper panel **(A–E)** shows a representative whole brain tissue section from the third coronal brain slice of a hu-BLT-hIL34 mouse (#1703) that was stained immunohistochemically for hIba-1+ (brown) and counterstained with hematoxylin. LV stands for lateral ventricle. The blue and red boxed regions in the Figure C were highlighted at a higher magnification **(A, D)**. In turn, the blue and red boxed regions of the **(A, B)** were further highlighted **(B, E)**. The hIba-1+ cells are morphologically ramified and mainly distributed in brain parenchyma. The lower panel **(F–J)** shows a representative whole brain tissue section **(H)** of a hu-BLT mouse (#1717) that was stained immunohistochemically for hIba-1+ cells (brown). The blue and red boxed regions in the Figure H was highlighted at a higher magnification **(F, I)**. In turn, the blue and red boxed regions of the **(F, I)** were further highlighted **(G, J)**. There were no detectable parenchymal hIba-1+ cells.

**Figure 3:**
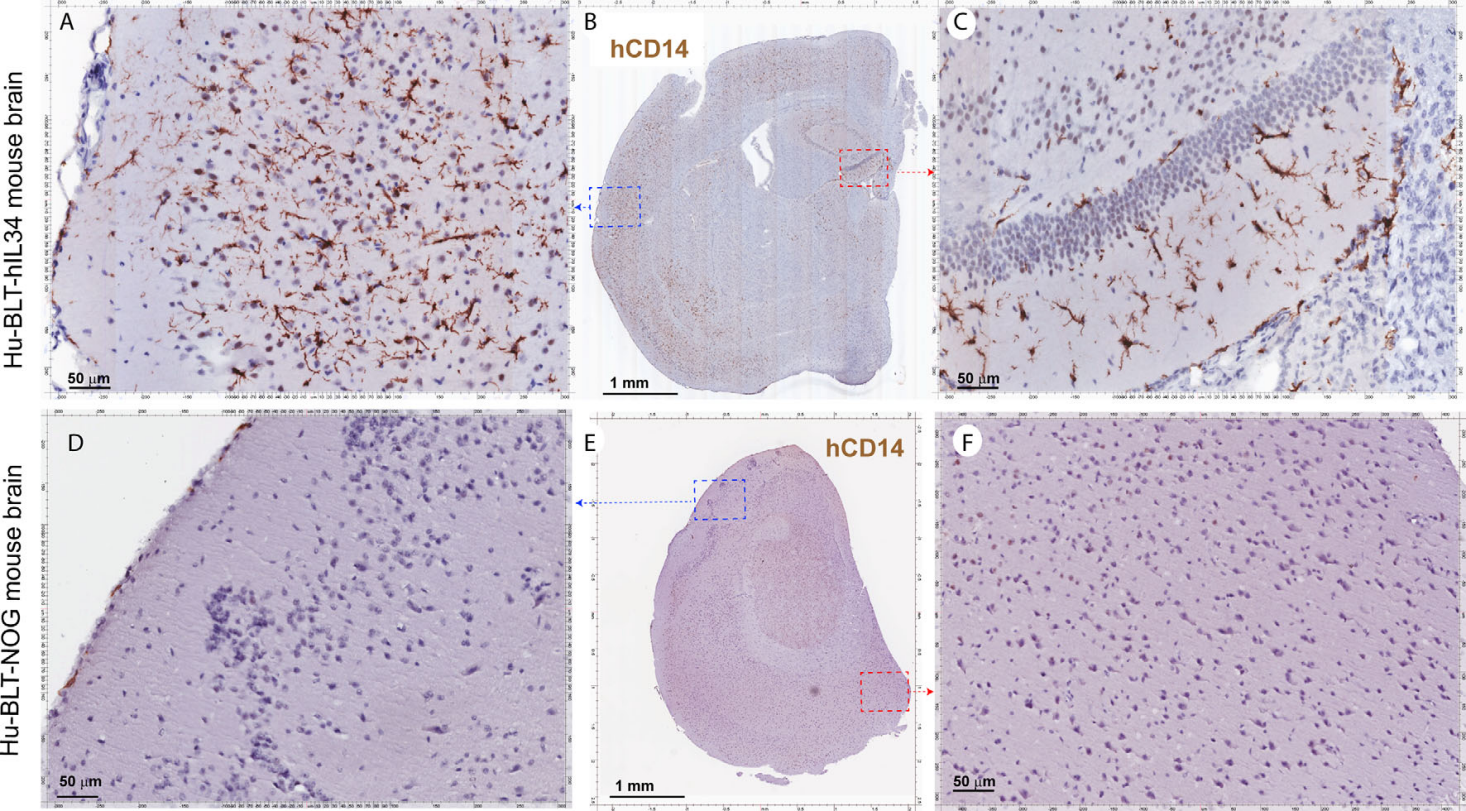
The hCD14+ myeloid cell reconstitutions in the brain tissues of hu-BLT-hIL34 BLT and hu-BLT mice. The upper panel **(A–C)** shows representative whole brain tissue sections and the highlighted images form the corresponding boxed regions from hu-BLT-hIL34 mouse (#1708) that were stained immunohistochemically for hCD14+ cells (brown) and counterstained with hematoxylin. There were abundant hCD14+ cells **(A–C)** in the brain of the hu-BLT-hIL34 mouse. The lower panel **(D–F)** in contrast shows no detectable hCD14+ myeloid cells in the brain parenchyma and a very limited number of hCD14+ cells in meninges in a representative whole brain tissue section of a hu-BLT mouse (#1723).

CNS contains four types of macrophages: parenchymal microglia and nonparenchymal perivascular macrophages (PVM), meningeal macrophages (MM) as well as choroid plexus macrophages (2, 8). First, we used antibodies to hIba-1, hCD14, hCD68 and hCD163, sensitive markers of macrophages and microglial cells (27–30), to evaluate human myeloid cells reconstitutions in the CNS of hu-BLT-hIL34 and hu-BLT mice. We examined human myeloid cells and microglial reconstitutions in the CNS of all six hu-BLT-hIL34 mice using IHCS with antibodies to hIBA-1, hCD14, hCD68, hCD163, and hTMEM119. To compare, we also evaluated the human myeloid cells and microglial reconstitution in 6 hu-BLT mice using IHCS with above antibodies. The frequency and distribution of human myeloid cells and microglial cells in CNS of these mice are highly consistent among these different antibodies. There were extensively reconstitutions of human myeloid cells in the CNS parenchyma of hu-BLT-hIL34 mice as indicated by hIba-1+ cells (**Figure 2** and **Supplementary Figure 1**), hCD14+ cells (**Figure 3** and **Supplementary Figure 2**), CD68+ cells (**Supplementary Figure 2**) and CD163+ cells (**Supplementary Figure 3**) using IHCS. As shown in a representative whole brain tissue section from the third coronary slice (**Figure 2C**) of all hu-BLT-hIL34 mouse (#1703), hIba-1+ cells in the brain parenchyma were extensively reconstituted across multiple regions of the brain. The **Figures 2A, D** respectively highlighted the cerebral cortex and hippocampus boxed regions from the **Figure 2C**; in turn, **Figures 2B, E** respectively further highlighted the boxed regions from the **Figures 2A, D** at a higher magnification. We also detected extensive reconstitution of hIba-1+ cells in spinal cord (**Supplementary Figures 1A, B**, mouse# 1708) and cerebellum tissues (**Supplementary Figures 1C, D**, mouse#1708) of hu-BLT-hIL34 mice. The hIba-1+ cells are numerous, morphologically ramified, distributed in brain parenchyma. Consistent with the extensive reconstitution of parenchymal human myeloid cells in the CNS revealed by hIba-1+ cells, there were also abundant hCD14+ myeloid cells as shown in a representative whole brain tissue sections (**Figures 3A–C**, mouse#1708) of hu-BLT-hIL34 mice. The blue and red boxed regions of cerebral cortex and hippocampus in the **Figure 3B** were respectively highlighted at a higher magnification in the **Figures 3A, C**. The hCD14+ cells are again morphologically ramified and mainly distributed in brain parenchyma. Consistent with the extensive reconstitutions of hIBa-1+ and hCD14+ cells above mentioned, there were also abundant hCD68+ cells (**Supplementary Figure 2**) and hCD163+ cells (**Supplementary Figure 3**) in the brain of hu-BLT-hIL34 mice. We thus concluded that hu-BLT-hIL34 mice extensively reconstituted parenchymal human myeloid cells in the CNS, including cerebral cortex, hippocampus, thalamus hypothalamus, striatum, amygdala, spinal cord, and cerebellum (**Figures 2** **,** **3** and **Supplementary Figures 1**–**3**). In contrast and as expected, there were an absence of detection or very limited reconstituted hIBa-1+ cells (**Figures 2F–J**, **Supplementary Figures 1E–H**), hCD14+ cells (**Figures 3D–F** and **Supplementary Figure 2**), hCD68+ cells (**Supplementary Figure 2**), or hCD163+ cells (**Supplementary Figures 3D–F**) cells in the CNS of the hu-BLT mice. As shown in a representative whole brain tissue section from the third coronal slice (**Figures 2F–J**, mouse#1717) of hu-BLT mice, hIba-1+ cells were absent in all the parenchyma of cerebral brain (**Figures 2F–J**). Similarly, hCD14+ cells were also absent in the parenchyma of hu-BLT mice (**Figures 3D–F**, mouse#1723). We next compare the reconstitution of nonparenchymal perivascular macrophages and meningeal macrophages in the hu-BLT-hIL34 and hu-BLT mice. As indicated by hIba-1, hCD14, hCD163 expression and their anatomic distribution, hu-BLT-hIL34 mice also have a better reconstitution of meningeal macrophages (MM) and perivascular macrophages (PVM) than hu-BLT mice (**Figures 2** **,** **3** and **Supplementary Figures 1**–**3**).

### The Myeloid Cells in Brain Parenchyma Expressed Microglia-Specific Marker hTMEM119

**Figure 4:**
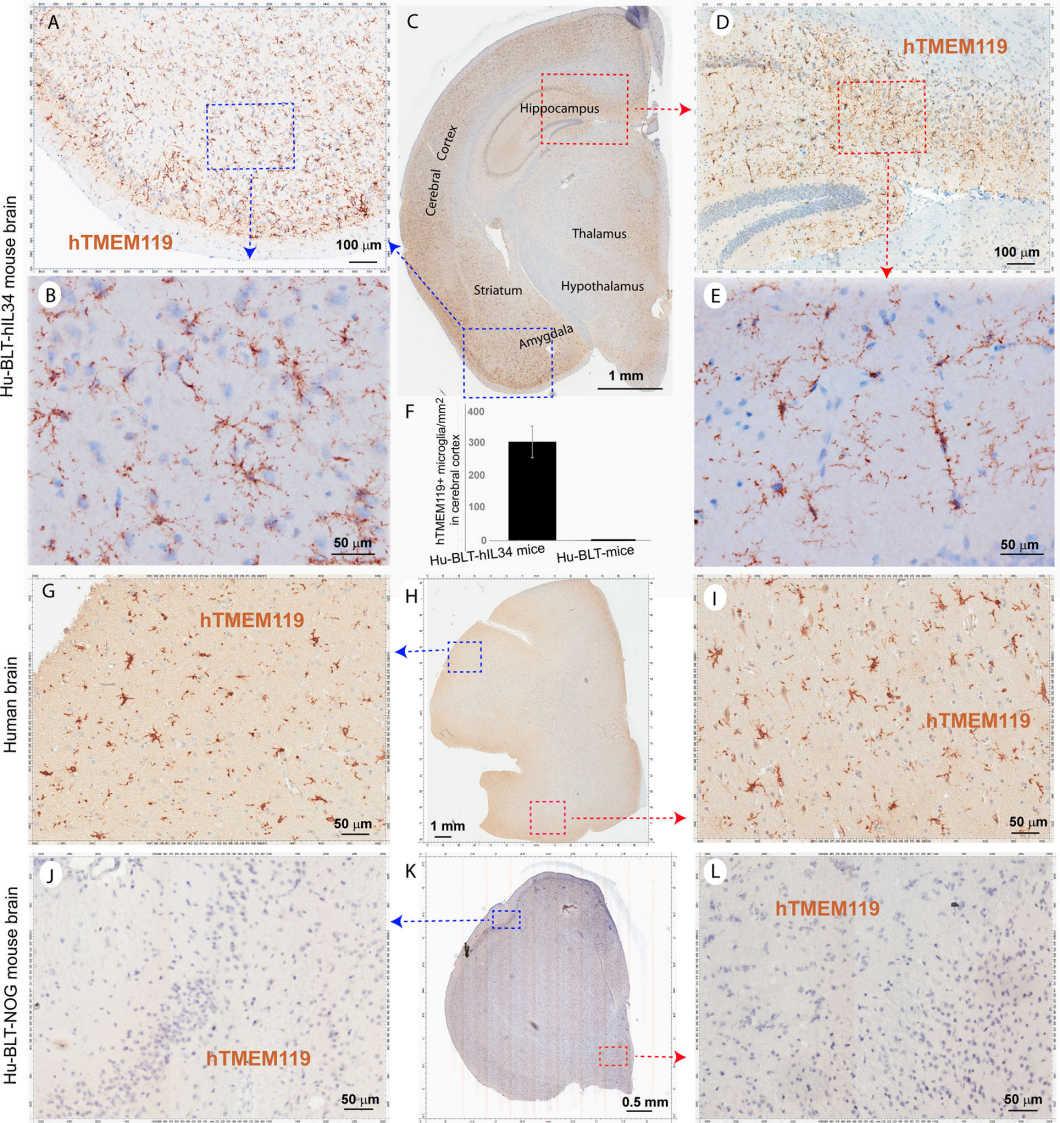
The microglia-specific marker hTMEM119+ microglia in the brain tissues of hu-BLT-hIL34 and hu-BLT mice and humans. A representative whole brain tissue section **(C)** from the third coronal brain slice of a hu-BLT-hIL34 mouse (#1703) that was stained immunohistochemically for hTMEM119+ cells (brown) and counterstained with hematoxylin. LV stands for lateral ventricle. The blue and red boxed regions in the Figure **(C)** were highlighted at a higher magnification **(A, D)**. In turn, the blue and red boxed regions of the Figures **(A, D)** were highlighted **(B, E)**. The hTMEME119+ cells (brown) are morphologically ramified and distributed in brain parenchyma. The histogram of quantitative images analysis of hTMEM119 + cells in the cerebral cortex of the hu-BTL-hIL34 mice **(F)** (304.08 ± 131.93 hTEME119+ cells/mm2, n=6), where there were an absent of these cells in the hu-BLT mice. The middle panel **(G–I)** shows hTMEM119 + microglia in the cerebral cortex of a HIV-1 non-infected individual with no registered medical complications **(G–I)**. The frequency, distribution, and morphology of hTMEM119+ human microglia in hu-BLT-hIL34 mice are similar to this human individual. The lower panel **(J–L)** shows no detectable hTMEM119+ cells in a representative whole brain section of a hu-BLT mouse (#1721).

To distinguish parenchymal microglia from macrophages, we conducted IHCS using microglial-specific marker hTMEM119 (31, 32). There were extensively reconstitutions of hTMEM119+ cells in the CNS parenchyma of hu-BLT-hIL34 mice (**Figures 4A–F**, mouse#1703). As shown in a representative whole brain tissue section from the third coronal slice (**Figure 4C**), hTMEM119+ cells in the brain parenchyma were extensively reconstituted across multiple regions of the brain. The **Figures 4A, D** respectively highlighted the boxed regions of the cerebral cortex and hippocampus from the **Figure 4C**; in turn **Figures 4B, E** respectively further highlighted the boxed regions from the **Figures 4A, D** at a higher magnification. The hTMEM119+ cells are morphologically ramified and distributed in parenchyma. We quantified hTMEM119 + cells in the cerebral cortex of the hu-BTL-hIL34 mice (n=6) and found there were 304.08 ± 131.93 (mean ± SD) hTMEM119+ microglial cells/mm2, whereas there were an absent of these cells in the hu-BLT mice. We further compared hTMEM119 + cells in the cerebral cortex of an HIV-1 non-infected individual, who had no registered medical complications at NIH Neuro Biobank. We found that the frequency, morphology, and distribution of hTMEM119+ cells in hu-BLT-hIL34 mice are similar to that person (**Figures 4G–I**). In contrast, there were an absence of detectable hTMEM119+ cells in the CNS of the hu-BLT mice (**Figures 4J–L**, mouse#1721). We thus concluded that hu-BLT-hIL34 mice extensively reconstituted human microglia in the CNS.

### HIV-1 Infection in the CNS of hu-BLT-hIL34 Mice

**Figure 5:**
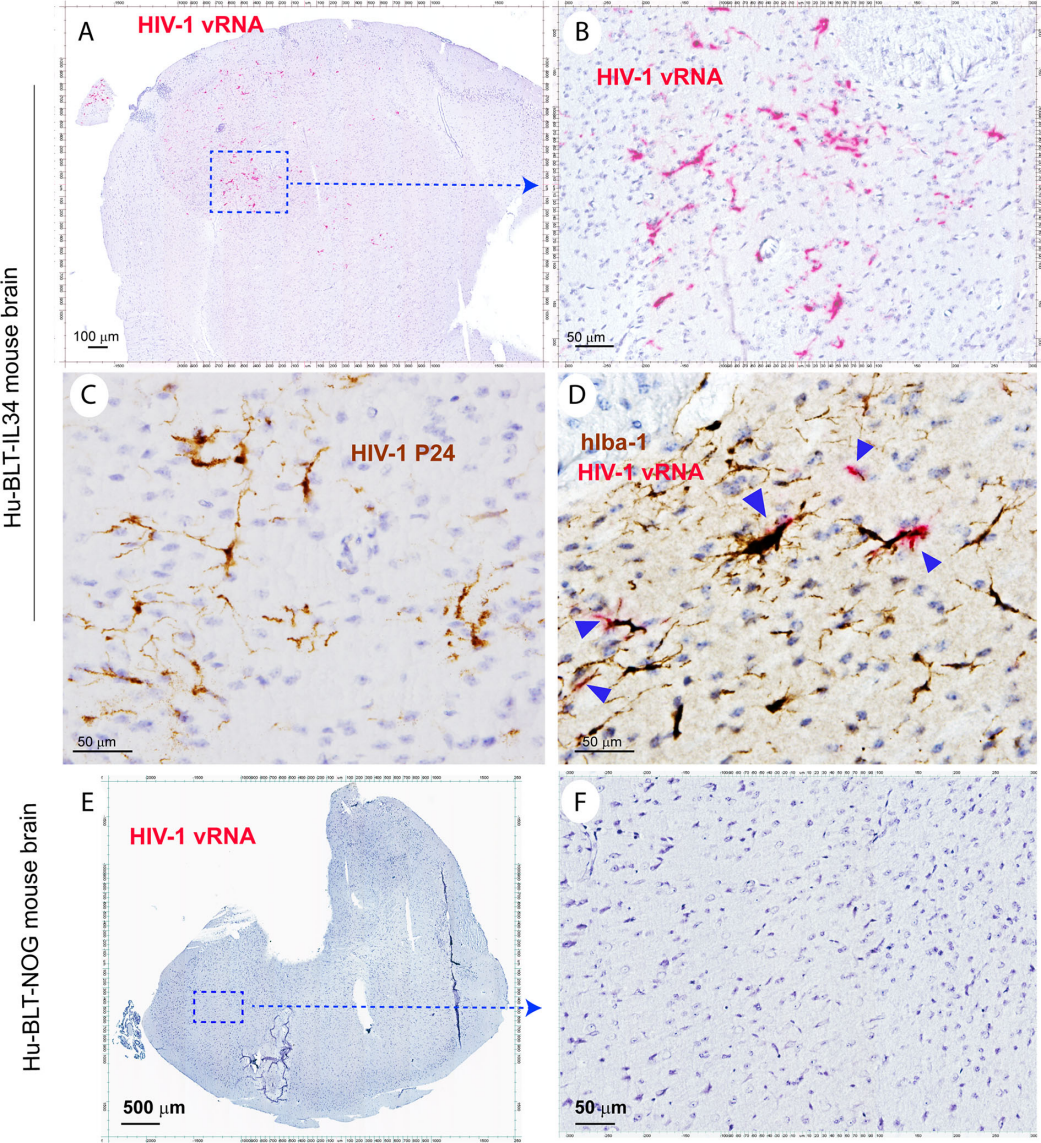
The HIV-1 infection of human microglia of hu-BLT-hIL34 mice. The Upper panel shows HIV-1 RNA+ cells (**A, B**, red) in a representative whole brain tissue section from a hu-BLT-hIL34 mouse (#1708) that was detected using RNAscope *in situ* hybridization with HIV-1 clade B anti-sense probe and counterstained with hematoxylin. The highlighted boxed region from the Figure **(A)** was shown at a higher magnification of HIV-1 RNA+ cells (**B**, red). HIV-1 p24+ cells (**C**, brown) in the cerebral cortex of the same mouse were detected using IHCS. The colocalization of HIV-1 vRNA and human myeloid cell marker hIba-1 (**D**, arrows) indicating the reconstituted human myeloid cells could support HIV-1 replication. The lower panel **(E, F)** shows there were no detectable HIV-1 RNA+ cells in a representative whole brain tissue section from a hu-BLT mouse (#1724) using RNAscope *in situ* hybridization.

To test the functionality of reconstituted human myeloid cells in the CNS of hu-BLT-hIL34 mice, we infected 4 animals from hu-BLT-hIL34 group and 5 animals from hu-BLT mice groups (**Supplemental Table 1**). At 4-6 weeks post HIV-1 infection, we euthanized all 4 hu-BLT-hIL34 mice that were inoculated with HIV-1 Ada. We detected abundant HIV-1 vRNA+ and p24+ cells in the brain tissues of all 4 hu-BLT-hIL34 mice that were inoculated with HIV-1 (**Figures 5A–D**, mouse#1708) but there were undetectable HIV-1 vRNA+ cells in three hu-BLT mice that were inoculated with HIV-1 and examined (**Figures 5E, F**, mouse#1724). As shown in representative images in the **Figure 5**, there were abundant HIV-1 RNA+ cells (red) in the brain tissues of hu-BLT-hIL34 mouse (#1708) detected using RNAscope ISH with HIV-1 clade B probe. Consistent with the results of HIV-1 VRNA+ cells, there were abundant HIV-1 p24+ cells detected using IHCS. We further defined the HIV-1 vRNA+ cells type as human myeloid cells using IHCS with hIba-1 marker (**Figure 5D**, arrows) indicating the reconstituted human myeloid cells can support HIV-1 infection. In contrast, we did not detect any HIV-1 vRNA+ cells in the brain of hu-BLT mice (**Figures 5E, F**, mouse#1724).

## Discussion

In this study, we demonstrated that human microglia can be extensively reconstituted in CNS from circulating human HSPCs in hu-BLT-hIL34 mice. We first used a battery of human myeloid cell markers, including hIBa-1, hCD14, hCD68 and hCD163, to evaluate human myeloid cell reconstitution in the CNS and found that human myeloid cells were extensively reconstituted and primarily localized in the brain parenchyma (**Figures 2** **,** **3** and **Supplementary Figures 1**–**3**). We then used a human microglial specific marker, hTMEM119, to validate these reconstituted human myeloid cells in the brain parenchyma are mainly human microglia (**Figures 4A–F**). Further, in comparison with hTMEM119 + microglia in the cerebral cortex of a HIV-1 non-infected individual with no registered medical complications (**Figures 4G–I**), we found the frequency, distribution, and morphology of hTMEM119+ human microglia in hu-BLT-hIL34 mice are similar to this person. Our data thus support the notion that human microglia at adults can be generated through human hematopoietic stem and progenitor cells (HSPC), which is consistent with the previous reports that bone marrow derived cells can enter brain to different into microglia at adults (9–11). In contrast, we did not observe human microglia reconstitution in the brain parenchymal of hu-BLT mice (**Figures 2**–**4** and **Supplementary Figures 1**–**3** lower panels). The hu-BLT-hIL34 and hu-BLT mice are genetically identical and also received the same human donor transplant except the former had hIL34 knock-in, indicating that hIL34, a ligand of the colony stimulating factor-1 receptor, play an important role in myeloid and microglial cells development in CNS (19, 33). This study is unique in the following aspects. First human myeloid and microglia cells reconstitution in the CNS is a clear-cut result in this chimeric mouse and human model. Second, the adult mice engrafted with HSPCs extensively reconstituted human microglia in the CNS, to our knowledge this is the first report in this regard. In addition to comparing the parenchymal human microglia between hu-BLT-hIL34 and hu-BLT mice, we also observed that hu-BLT-hIL34 mice had a much better reconstitution of meningeal and perivascular macrophages than hu-BLT mice (**Figures 2** **,** **3** and **Supplementary Figure 3**). We also would like to point that both hu-BLT-hIL34 and hu-BLT mice received sublethal irradiation, whether this irradiation facilitated HSPCs to gain an entry into the brain and whether without irradiation can also reconstitute the human brain microglia in adult hIL34-NOG mice remains to be investigated.

Despite the importance of microglial cells, as a resident macrophage, in host immune response to brain infections and in the pathogenesis of neurodegenerative diseases, very limited small animal models are available to recapitulate diseases pathogenesis associated with human microglia. To that end, we infected hu-BLT-hIL34 mice and found that reconstituted human microglia are susceptible to HIV-1 infection in the CNS. Moreover, HIV-1 vRNA were localized in human myeloid cells in the brain, reenforcing that microglia cells are the most important subtract of HIV-1 infection in the CNS (26, 34). Using hu-BLT-hIL34 mice, it is now feasible to investigate the interplay between human pathogens, such as HIV-1, with human immune system of periphery and CNS compartments. The hu-BLT-hIL34 mouse model reported here open a new avenue for investigating the pathogenesis of HIV-1 infection and purging HIV-1 latent reservoir in the CNS in addition to peripheral tissues.

## Data Availability Statement

The original contributions presented in the study are included in the article/**Supplementary Material**. Further inquiries can be directed to the corresponding authors.

## Ethics Statement

Ethically sourced human autopsy cerebral cortex tissues from a deidentified individual of HIV-1 negative with no registered medical complications were obtained from the NIH Neuro BioBank (https://neurobiobank.nih.gov/). The patients/participants provided their written informed consent to participate in this study. All methods associated with animals described in this study were conducted in accordance with the Institutional Animal Care and Research Committee (IACUC) approved protocols at the University of Nebraska-Lincoln (UNL) and University of Nebraska Medical Center (UNMC).

## Author Contribution

QL and JZ designed the experiments and wrote the manuscript. SG and LG bred the mice. JZ, SL, and YC generated hu-BLT mice and conducted animal HIV-1 infection experiment. JZ performed immunohistochemical staining and *in situ* hybridization on animal tissues and W-KK did the human brain tissue immunohistochemical staining. TW did the quantification of hTMEM119 + cells. All authors contributed to the article and approved the submitted version.

## Funding

This study is supported in part by the National Institutes of Health (NIH) Grants P30 MH062261-16A1 Chronic HIV Infection and Aging in NeuroAIDS (CHAIN) Center (to Buch & Fox), R21 AI143405 (to QL). R01 AI136756 (YL, QL). The funders had no role in study design, data collection and analysis, preparation of the manuscript, or decision for publication.

## Conflict of Interest

The authors declare that the research was conducted in the absence of any commercial or financial relationships that could be construed as a potential conflict of interest.

## Supplementary Material

Supplementary Figure 1 The hIba-1+ myeloid cell reconstitutions in the spinal cord and cerebellum tissues of hu-BLT-hIL-34 and hu-BLT mice. The upper panel shows abundant hIba-1+ cells (brown) from representative spinal cord (**A, B**, mouse# 1708) and cerebellum (**C, D**, mouse# 1703) tissue sections of a hu-BLT-hIL34 mice detected using IHCS. The lower panel shows no detectable hIba-1+ cells (brown) from representative spinal cord (**E, F**, mouse# 1721) and cerebellum (**G, H**, mouse#1717) tissue sections of a hu-BLT mice detected using IHCS.

Supplementary Figure 2 The comparison of human myeloid cell reconstitutions in the cortex, corpus striatum, hippocampus, and cerebellum tissues of hu-BLT-hIL-34 and hu-BLT mice. Every upper panel respectively shows human myeloid lineage cells of hIba-1+, hCD14+, hCD68+ or hTMEM119+ were extensively reconstituted throughout the cortex, corpus striatum, hippocampus and cerebellum of brains of hu-BLT-hIL34 mice; while every lower panel respectively shows an absence of or limited detection of these cells from hu-BLT mice using IHCS. Each amplified micrograph was shown from the red frame in the left upper corner thumbnails pictures. Scale bar equals 25μm.

Supplementary Figure 3 The comparison of human meningeal macrophages (MM) and perivascular macrophages (PVM) reconstitutions in the brain tissues of hu-BLT-hIL-34 and hu-BLT mice. The upper panel shows abundant hCD163+ (brown) human parenchymal myeloid cells **(A–C)**, human meningeal macrophages (**A**, arrows) and human perivascular macrophages **(C)** from representative brain tissue sections of a hu-BLT-hIL34 mice (#1708) detected using IHCS. The lower panel shows an absence of hCd163+ human parenchymal myeloid cells and a few of human meningeal macrophages (**D–F**, arrows) of hu-BLT mice (mouse#1726).
