# Supplementary figures and images for: Human Microglia Extensively Reconstitute in Humanized-BLT Mice With Human Interleukin-34 Transgene and Support HIV-1 Brain Infection
Source: Front Immunol. 2021 May 21;12:672415. doi: 10.3389/fimmu.2021.672415 (PMC8176960; doi:10.3389/fimmu.2021.672415)

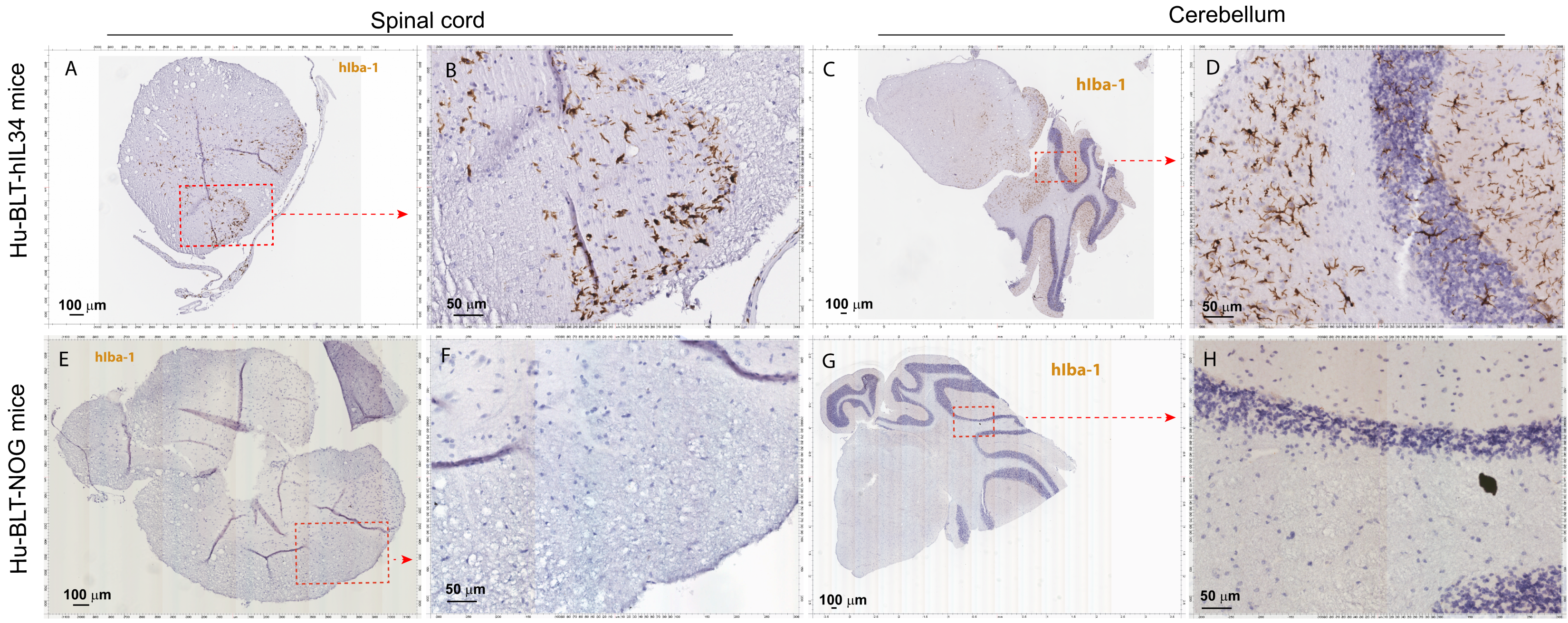

Supplement: Supplementary Figure 1 — The hIba-1+ myeloid cell reconstitutions in the spinal cord and cerebellum tissues of hu-BLT-hIL-34 and hu-BLT mice. The upper panel shows abundant hIba-1+ cells (brown) from representative spinal cord (A, B, mouse# 1708) and cerebellum (C, D, mouse# 1703) tissue sections of a hu-BLT-hIL34 mice detected using IHCS. The lower panel shows no detectable hIba-1+ cells (brown) from representative spinal cord (E, F, mouse# 1721) and cerebellum (G, H, mouse#1717) tissue sections of a hu-BLT mice detected using IHCS. [file Image_1.tif]

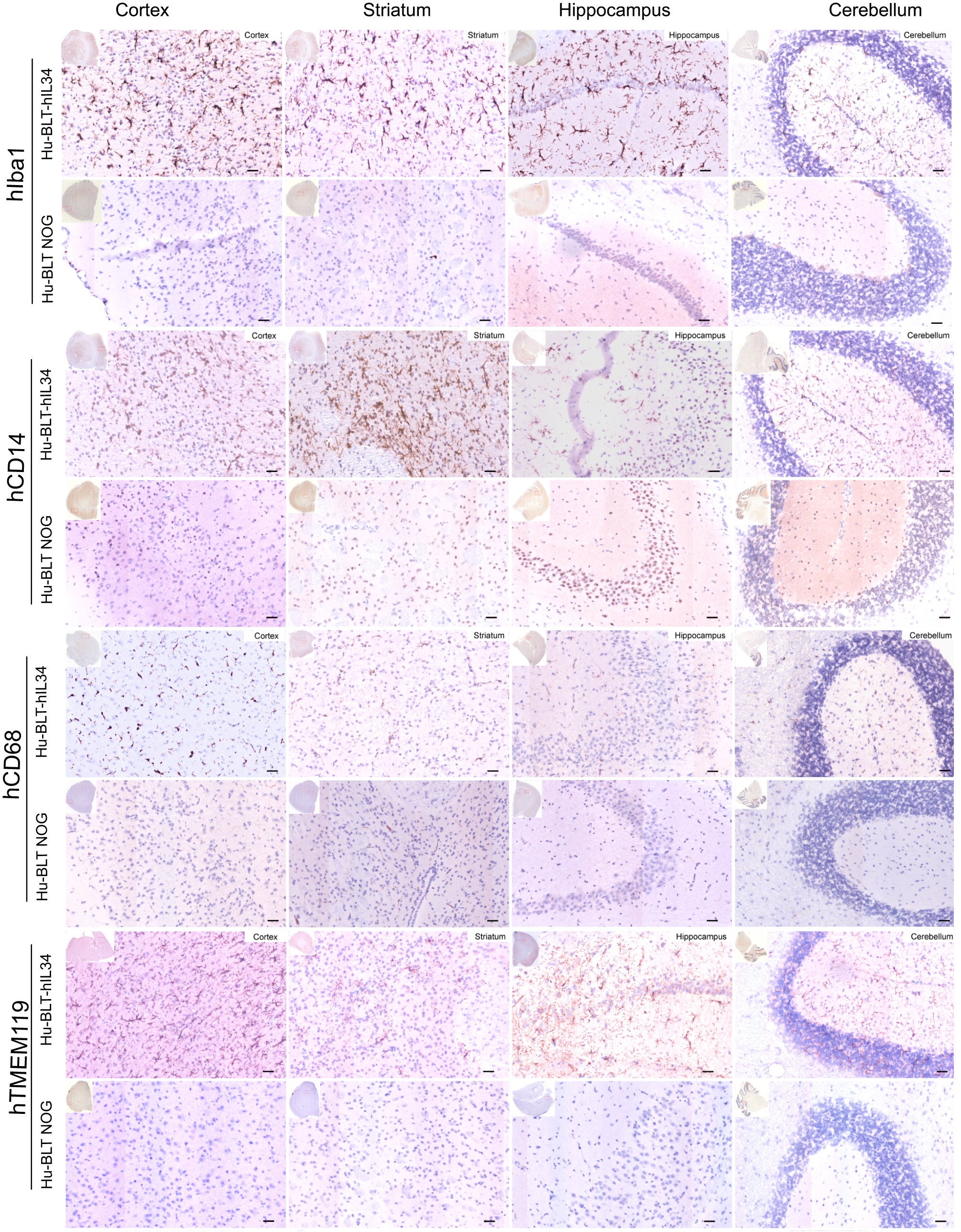

Supplement: Supplementary Figure 2 — The comparison of human myeloid cell reconstitutions in the cortex, corpus striatum, hippocampus, and cerebellum tissues of hu-BLT-hIL-34 and hu-BLT mice. Every upper panel respectively shows human myeloid lineage cells of hIba-1+, hCD14+, hCD68+ or hTMEM119+ were extensively reconstituted throughout the cortex, corpus striatum, hippocampus and cerebellum of brains of hu-BLT-hIL34 mice; while every lower panel respectively shows an absence of or limited detection of these cells from hu-BLT mice using IHCS. Each amplified micrograph was shown from the red frame in the left upper corner thumbnails pictures. Scale bar equals 25μm. [file Image_2.tif]

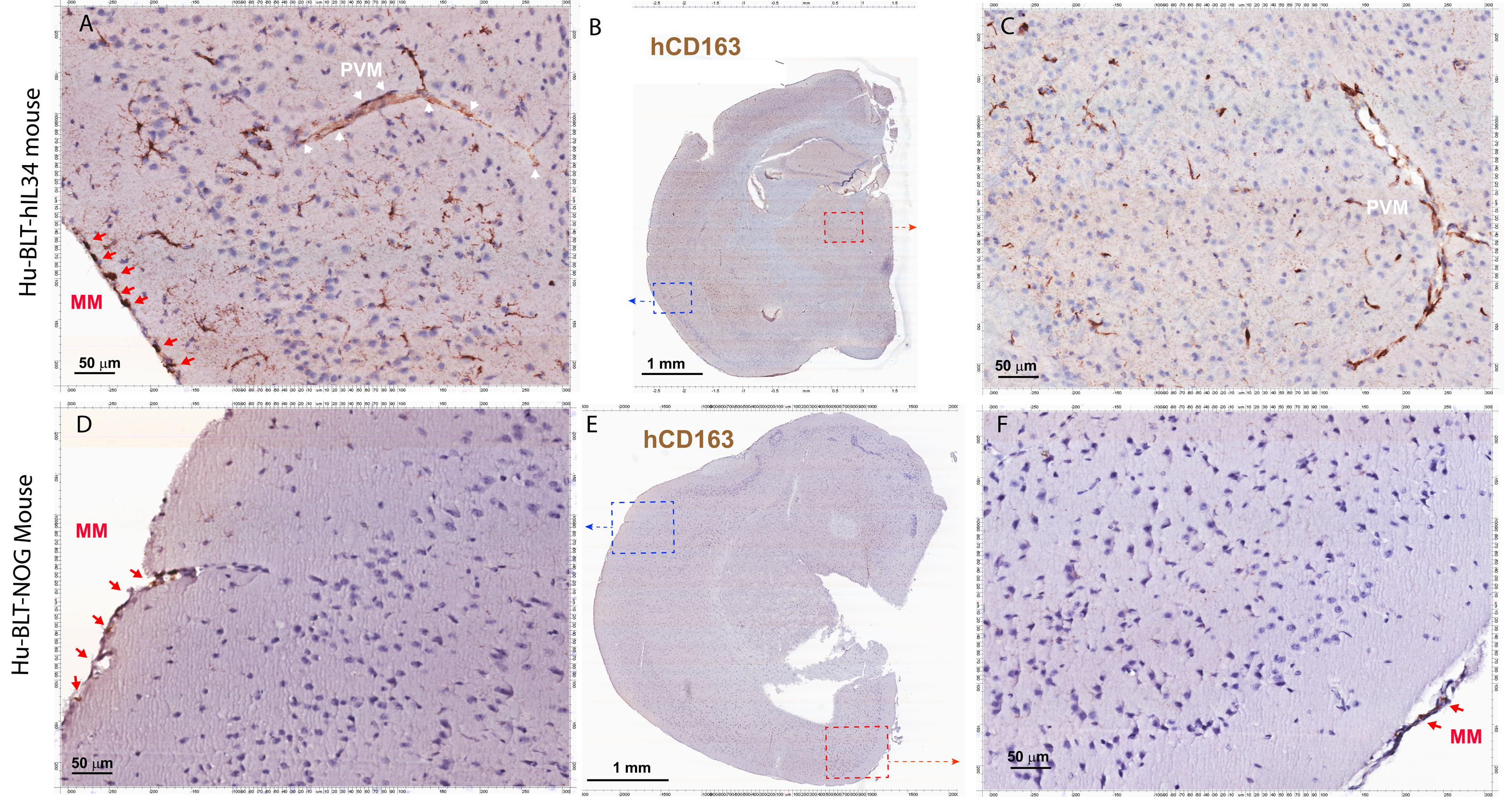

Supplement: Supplementary Figure 3 — The comparison of human meningeal macrophages (MM) and perivascular macrophages (PVM) reconstitutions in the brain tissues of hu-BLT-hIL-34 and hu-BLT mice. The upper panel shows abundant hCD163+ (brown) human parenchymal myeloid cells (A–C), human meningeal macrophages (A, arrows) and human perivascular macrophages (C) from representative brain tissue sections of a hu-BLT-hIL34 mice (#1708) detected using IHCS. The lower panel shows an absence of hCd163+ human parenchymal myeloid cells and a few of human meningeal macrophages (D–F, arrows) of hu-BLT mice (mouse#1726). [file Image_3.tif]
