# Supplementary material for: Human Microglia Extensively Reconstitute in Humanized-BLT Mice With Human Interleukin-34 Transgene and Support HIV-1 Brain Infection
Source: Front Immunol. 2021 May 21;12:672415. doi: 10.3389/fimmu.2021.672415 (PMC8176960; doi:10.3389/fimmu.2021.672415)
Supplement: Supplementary file 4 [file Table_1.docx]

| **Supplemental Table 1** HIV-1 infection of hu-BLT-hIL34 and hu-BLT mice | | | |
| --- | --- | --- | --- |
| **Group** | **Sub-group** | **Mouse ID** | **PVL, 4 wks PI** |
| Hu-BLT-hIL34 Mice | HIV-1 infection | 1703 | 8.45E+05 |
|  |  | 1705 | 1.09E+06 |
|  |  | 1708 | 4.56E+06 |
|  |  | 1709 | 1.36E +05 |
|  | Un-infection | 1699 | n/a |
|  |  | 1707 | n/a |
| Hu-BLT Mice | HIV-1 infection | 1720 | 8.28E+05 |
|  |  | 1723 | 5.78E+05 |
|  |  | 1724 | 1.09E+06 |
|  |  | 1726 | 1.36E+06 |
|  |  | 1728 | 5.46E+06 |
|  | Un-infection | 1717 | n/a |
|  |  | 1718 | n/a |
|  |  | 1721 | n/a |
|  |  | 1722 | n/a |
|  |  | 1729 | n/a |
